# Actions of the TrkB Agonist Antibody ZEB85 in Regulating the Architecture and Synaptic Plasticity in Hippocampal Neurons

**DOI:** 10.3389/fnmol.2022.945348

**Published:** 2022-06-30

**Authors:** Charlotte Tacke, Peter S. DiStefano, Ronald M. Lindsay, Kristin Metzdorf, Marta Zagrebelsky, Martin Korte

**Affiliations:** ^1^Division of Cellular Neurobiology, Zoological Institute, Technical University of Braunschweig, Braunschweig, Germany; ^2^Zebra Biologics, Inc., Concord, MA, United States; ^3^Helmholtz Centre for Infection Research, Research Group Neuroinflammation and Neurodegeneration (AG NIND), Braunschweig, Germany

**Keywords:** neurotrophins, TrkB receptor, dendritic spines, LTP, plasticity, neuronal architecture, TrkB agonist, BDNF

## Abstract

Signaling of BDNF *via* its TrkB receptor is crucial in regulating several critical aspects of the architecture and function of neurons both during development and in the adult central nervous system. Indeed, several neurological conditions, such as neurodevelopmental and neurodegenerative disorders are associated with alterations both in the expression levels of BDNF and TrkB, and in their intracellular signaling. Thus, the possibility of promoting BDNF/TrkB signaling has become relevant as a potential therapeutic intervention for neurological disorders. However, the clinical potential of BDNF itself has been limited due to its restricted diffusion rate in biological tissue, poor bioavailability and pharmacological properties, as well as the potential for unwanted side effects due to its ability to also signal *via* the p75^NTR^ pathway. Several small molecule and biologic drug candidate TrkB agonists have been developed and are reported to have effects in rescuing both the pathological alterations and disease related symptoms in mouse models of several neurological diseases. However, recent side-by-side comparative studies failed to show their specificity for activating TrkB signaling cascades, suggesting the need for the generation and validation of improved candidates. In the present study, we examine the ability of the novel, fully human TrkB agonist antibody ZEB85 to modulate the architecture, activity and synaptic plasticity of hippocampal murine neurons under physiological conditions. Moreover, we show here that ZEB85 prevents β-amyloid toxicity in cultured hippocampal neurons, in a manner which is comparable to BDNF.

## Introduction

Brain-derived neurotrophic factor (BDNF) plays a pivotal role in establishing and maintaining the structure and function of neurons in the central nervous system, signaling *via* its receptor tropomyosin receptor kinase B (TrkB) (Poo, 2001; Minichiello, 2009; Cohen-Cory et al., 2010; Zagrebelsky and Korte, 2014). BDNF/TrkB signaling regulates the fine-tuning of the neuronal network during development by controlling neuronal differentiation, dendritic development and synaptogenesis (Cohen-Cory et al., 2010; Park and Poo, 2013). Moreover, BDNF is involved in the maintenance of neuronal architecture including number, size and shape of dendritic spines (Kellner et al., 2014; Zagrebelsky et al., 2020), and is crucial for synaptic plasticity (Zagrebelsky and Korte, 2014; Kowianski et al., 2018), as well as learning and memory processes (Tyler et al., 2002; Cunha et al., 2010). Transport, synthesis and secretion of BDNF occur in an activity-dependent manner (Aicardi et al., 2004; Tongiorgi, 2008) providing spatially and temporally precise actions at synapses (Harward et al., 2016). Perturbations in any of these BDNF/TrkB-mediated functions underlie the pathogenesis of several neurological conditions, as suggested by the reduced BDNF/TrkB signaling in neurodegenerative, neurodevelopmental, and psychiatric diseases (Zuccato and Cattaneo, 2007; Gupta et al., 2013; Castrén and Monteggia, 2021). Although, treatment with recombinant BDNF is beneficial in animal models of different neurological diseases (Chang et al., 2006; Nagahara et al., 2009; Zuccato and Cattaneo, 2009; Jiao et al., 2016; de Pins et al., 2019), clinical trials with BDNF have generally been disappointing. Poor bioavailability, due to small molecular size and highly basic charge, are supposed to limit the clinical utility of BDNF (Lu et al., 2013; Palasz et al., 2020). In addition, exogenously applied BDNF may exert unwanted effects by binding to the p75 neurotrophin receptor (p75^NTR^) – sortilin complex (Woo et al., 2005) mediating opposite cellular functions than TrkB (Friedman, 2000; Chapleau and Pozzo-Miller, 2012). One approach to overcome the poor pharmacological properties of BDNF, is the development of highly selective TrkB agonists. Small-molecule compounds specifically acting on TrkB (Jang et al., 2010; Massa et al., 2010) show efficacy in several animal models of neurological diseases (Simmons et al., 2013; Chen et al., 2018; Ren et al., 2019). However, two independent side-by-side comparisons of these small molecules could not detect activation of TrkB or its intracellular signal transduction pathway despite their positive behavioral effects (Todd et al., 2014; Boltaev et al., 2017), raising doubts about their specificity and potential therapeutic use. Several TrkB agonist antibodies have been identified as BDNF mimetics and shown to induce receptor activation consistent with the activation profile upon binding of BDNF (Qian et al., 2006; Todd et al., 2014; Merkouris et al., 2018; Guo et al., 2019). The recently identified fully human TrkB agonist antibody ZEB85 (Merkouris et al., 2018) very closely mimics the BDNF with regard to TrkB binding and canonical phosphorylation signaling in human embryonic stem cell-derived neurons. Moreover, ZEB85 has been shown to be highly selective for TrkB since it does not recognize nor activate TrkA and TrkC, and does not bind to p75^NTR^ (Merkouris et al., 2018). ZEB85 also activates TrkB in cultured mouse cortical neurons, albeit with less potency. Similar to BDNF, ZEB85 promotes the maintenance of dendritic arbors of axotomized adult mouse retinal ganglion cells in explant cultures (Merkouris et al., 2018), an established BDNF/TrkB signaling-dependent assay (Binley et al., 2016).

In the present study, we examined the ability of ZEB85 to modulate the structure and plasticity of primary hippocampal murine neurons under physiological conditions. Moreover, we tested whether application of ZEB85 may rescue the deleterious structural alterations in hippocampal neurons observed upon exposure to toxic β-amyloid_1–42_.

## Materials and Methods

### Animals

All mice used in this study were maintained on a C57Bl/6J genetic background and were bred in the mouse facility of the TU Braunschweig. The mice were housed on a 12 h light/dark cycle in a temperature-controlled environment (21 ± 2°C) with food and water provided *ad libitum*. All experimental procedures were approved by the animal welfare representative of the TU Braunschweig and the LAVES (Oldenburg, Germany, Az. §4 (02.05) TSchB TU BS).

Mice carrying two floxed *bdnf* alleles (*bdnf^lox/lox^*) were created in the group of Yves-Alain Barde by inserting LoxP sites flanking exon IX (5′ loxP at bp 906, 5′ loxP at bp 3449 locus AY057907), the single protein coding exon of *bdnf* (Rauskolb et al., 2010). The heterozygous BDNF-knockout (BDNF-KO) mouse was generated by replacing a 560 bp fragment from the BDNF protein-coding exon with a selection marker, thus deleting most of the mature BDNF coding sequence (Korte et al., 1995). Genotypes of mice were assessed by PCR using genomic DNA purified from the tail as previously described (Rauskolb et al., 2010).

### Reagents and Stock Solutions

The TrkB agonist antibody ZEB85 and the control-antibody (GGVV-FC) were produced as previously described (Merkouris et al., 2018). For ZEB85, the ectodomain of human TrkB (through a.a. 432 – up to the transmembrane domain) was used as the antigen that was panned with a scFv recombinant human antibody library to generate ZEB85. The GGVV antibody was raised against a viral antigen using the same panning approach.

Recombinant Human BDNF protein (R&D systems) was dissolved in sterile PBS with 0.1% BSA at a stock concentration of 500 nM. The BDNF stock solution and the antibodies were stored at –70°C until they were used.

### Aβ Oligomer Preparation

The stock solution of soluble Amyloid-β_1–42_ (Aβ_1–42_) oligomers (oAβ) was prepared as previously described (Takata et al., 2003). Briefly, the lyophilized 1,1,1,3,3,3-hexafluoro-2-propanol (HFIP)-treated human Aβ_1–42_ peptides (Bachem) were dissolved in dimethylsulfoxide (DMSO; Invitrogen), sonicated for 20 min, aliquoted, and stored at –70°C. The Aβ_1–42_ peptides, dissolved in DMSO, were diluted in PBS to a final concentration of 33.5 μM and incubated at 4°C for 24 h to allow for oligomerization. The control contained no peptide but an equal amount of DMSO and was also incubated in PBS at 4°C for 24 h.

### Primary Hippocampal Cultures

Mouse hippocampal primary cultures were prepared at embryonic day 18 as previously described (Kellner et al., 2014). Briefly, embryos were rapidly decapitated, and the brains were immersed in ice cold Gey’s Balanced Salt Solution (GBSS) supplemented with glucose and adjusted to pH 7.2. The dissected hippocampi were incubated in Trypsin/EDTA (Sigma-Aldrich) at 37°C for 30 min after which the digestion was stopped. Subsequently, the neurons were dissociated mechanically using a Pasteur pipette and were re-suspended in Gibco Neurobasal medium supplemented with 2% B27, 10% N2 and 0.5 mM L-Glutamine and plated at different densities depending on the experiment performed: a high density (7 × 10^4^cells/cm^2^; analysis of the architecture of mature neurons and expression of cFos and pERK), middle density (3.5 × 10^4^ cells/cm^2^, measure of TrkB phosphorylation levels), low density (1 × 10^4^cells/cm^2^, analysis of the architecture in developing neurons) on poly-L-lysine-coated coverslips (12 mm, Sigma-Aldrich). The cultures were incubated at 37°C, 5% CO_2_, and 99% humidity. Once a week 20% of the medium was exchanged. Cultures were used at DIV7 or at DIV21/22.

### Transfection of Primary Hippocampal Cultures

Cultured hippocampal neurons were transfected at DIV20 with a plasmid encoding a membrane targeted farnesylated form of the enhanced green fluorescent protein (feGFP, Clontech) or with the red fluorescent monomeric derivative of DsRed pmApple-N1 (mApple) using Lipofectamine2000 (Thermo Fisher Scientific) according to the manufacturer’s protocol. Briefly, 0.8 μg feGFP or mApple and 2 μl Lipofectamine2000 were mixed in 100 μl Neurobasal medium, calculated per well, and added to the cultures in Neurobasal medium without supplements. The cultures were incubated for 50 min and afterwards the transfection medium was replaced with the original, previously collected, culture medium. Pharmacological treatments were performed 24 h later.

### Pharmacological Treatments

ZEB85 and the control-antibody were used at a final concentration of 10 nM and/or 100 nM when diluted in cell culture medium. BDNF was used as a positive control in some of the experiments at a final concentration of 1.64 nM and an equal volume of solvent (PBS with 0.1% BSA) was used as a control, except for the analysis of cFOS and pERK1/2 expression where only the control antibody was used. The different treatments were performed as follows:

(1) Developing primary hippocampal neurons were treated with control or ZEB85 antibody from DIV3 to DIV7 in fresh Neurobasal medium supplemented with B27 and N2 supplements.

(2) For the analysis of cFOS expression in mature hippocampal neurons (DIV21) the control or ZEB85 antibody was applied for 2 or 24 h and for pERK and pTrkB expression for 15 min in Neurobasal medium.

(3) Dendritic spine density and morphology were analyzed from DIV21-22 neurons after a 24 h application of control or ZEB85 antibody in Neurobasal medium. In a set of experiments a 15 min pre-treatment with control and ZEB85 antibody (100 nM) in Neurobasal medium was followed by the application of oligomerized Aβ_1–42_ or the respective control (equal volume of DMSO in PBS) added directly into the culture medium at a final concentration of 500 nM for 6 h.

(4) Primary hippocampal neurons derived from *bdnf^lox/lox^* mice transduced, or not with an AVV-CRE-GFP (Addgene Catalog#: 68544) at the day of preparation (DIV0) were treated with control or ZEB85 antibody (100 nM) starting on the same day in culture medium and then once a week until DIV21.

After the respective treatments the hippocampal cultures were fixed with paraformaldehyde (PFA; 4% PFA in 0.1 M phosphate buffer) for 10 min at room temperature and washed several times with PBS.

### Immunocytochemistry

The fixed cultured neurons were permeabilized with 0.2% Triton X-100 (Sigma-Aldrich) and any non-specific antibody binding sites were blocked with 1.5% Normal Goat Serum (Thermo Fisher Scientific) in PBS for 1 h at room temperature (RT). Subsequently, the neurons were incubated with primary antibodies diluted in 0.2% Triton X-100 and 1.5% Normal Goat Serum in PBS overnight at 4°C. The following primary antibodies were used: mouse anti-MAP2 (1:1000, Sigma-Aldrich), rabbit anti-cFos (1:10000, Synaptic systems), rabbit anti-Phospho-MAPK/Erk1/2 (1:1000, Cell Signaling Technology), rabbit Phospho-TrkA (1:500, Tyr674/675/TrkB; Tyr706/707;C50F3, Cell Signaling Technology), rabbit anti-parvalbumin (1:5000, Swant). The cultures were then washed with PBS and incubated in secondary antibodies diluted 1:500 in PBS for 2 h at RT. The following secondary antibodies were used: goat anti-rabbit IgG, goat anti-mouse IgG conjugated with appropriate cyanine fluorophores Cy2, Cy3 or Cy5 (Jackson ImmunoResearch Labs). After washing with PBS, the coverslips were mounted onto glass slides using anti-fading Fluoro-Gel embedding medium (Electron Microscopy Sciences) and stored in the dark at 4°C until they were imaged.

### Image Acquisition and Analysis

Image acquisition was performed using a Zeiss Axio Imager M2 microscope equipped with an ApoTome.2 module and a Zeiss Axiocam 702 mono camera (Carl Zeiss AG). The analysis was performed blind to the treatment.

### Imaging and Analysis of Neuronal Neurite / Dendritic Complexity

Isolated neurons labeled either with MAP2 (DIV7) or parvalbumin (DIV21-22) were imaged using a 20x (0.8 NA) objective. The neurons were traced using the Neurolucida 9 software (MicroBrightField) and analyzed with the Neurolucida explorer software (MicroBrightField). Neuronal complexity was assessed by analyzing total length, the number of primary processes, the number of branching points and the Sholl analysis (Sholl, 1953) to quantify complexity at 10 μm incremental intervals starting from the soma.

### Analysis of Dendritic Spine Density and Spine Type Distribution

Second or third order dendritic branches of feGFP-expressing DIV21-22 neurons, at least 60 μm long were imaged with a 63x (1.4 NA, oil) objective using the ApoTome.2 module to acquire 3D-stacks at 0.25 μm intervals. Spine density was calculated by dividing the number of dendritic spines by the length of dendritic segment (spines/μm), analyzed using the multipoint and the segmented line tools of ImageJ (National Institutes of Health). For the dendritic spine type distribution the spine length (from the spine base at the dendrite to spine tip), spine head width (at the largest diameter) and spine neck width (at the thinnest diameter) were measured using the segmented line tool of ImageJ and used to categorize them into different subtypes, as previously described (Zagrebelsky et al., 2005).

### Analysis of TrkB Phosphorylation by Immunofluorescence

TrkB activation was analyzed for the TrkB phosphorylation site Tyr706/707 (pTrkB). Primary isolated dendrites from MAP2 labeled hippocampal neurons were randomly chosen and imaged with a 63x (1.4 NA, oil) objective. The exposure time for the pTrkB and MAP2 fluorescence, set to the control, was kept constant across different treatments within the same experimental repetition. Non-specific background fluorescence was calculated for each neuron individually by placing small regions of interest (ROIs) in ImageJ along the dendrite in areas with no visible pTrkB fluorescence and by averaging the mean gray value of the ROIs (at least 15) per dendrite. For the analysis SynPAnal (Danielson and Lee, 2014) was used after subtraction of 2x background.

### Canonical Signal Transduction

cFos and pERK immunolabelled neurons were imaged with a 10x (NA 0.3) objective. Fields of view with a similar neuronal density were selected solely based on the MAP2 staining. The exposure time for each excitation wavelength was kept constant across different treatments within the same experimental repetition. For the analysis of cFOS/pERK positive neurons, a threshold was set based on the average fluorescent intensity of 10 neurons treated with the controls (0.1% BSA or control-antibody) and the number of positive neurons was counted using the multipoint tool of ImageJ. The percentage of cFOS/pERK positive neurons was calculated by dividing the number of cFOS/pERK positive neurons by the number of MAP2 positive neurons per field of view.

For the pERK and the 24 h cFos immunostaining the experiments using the two different concentrations of ZEB85, the relative control-antibody concentrations and BDNF were performed separately. However, since no statistically significant differences could be shown between the two sets for the two control-antibody concentrations and the BDNF, these results were pooled.

### Calcium Imaging

After a 24 h treatment with either ZEB85 antibody (100 nM) or BDNF and the respective controls, Ca^2+^ Imaging was performed in DIV20-25 primary mouse hippocampal neurons loaded with the chemical Ca^2+^ indicator Oregon-Green BAPTA-1 (OGB; Invitrogen). For dye loading, 5 μM OGB (stock solution 1 mM in DMSO) and a solution of 2.5% (w/v) Pluronic F-127 (Sigma-Aldrich) in DMSO were added to the Hank’s balanced salt solution (HBSS containing 50 ml HBSS (10x; Gibco), 175 mg NaHCO_3_, 147 mg CaCl_2_*2H_2_O, 1351 mg Glucose, total volume of 500 ml).

Each coverslip was incubated in the dye solution at 37°C, 5% CO_2_ and 99% humidity for 25 min. Subsequently, the coverslip was washed to remove extra dye and was transferred into an imaging chamber filled with HBSS at RT for 20 min for acclimation. A peristaltic pump was used to achieve a continuous flow of HBSS solution at a constant rate of 0.8 ml/min.

The dye-loaded neurons were imaged using a 40x objective (LUMPLFLN W NA 0.7) on an Olympus fluorescence Microscope BX61WI, equipped with a CCD camera (VisiCam QE, Visitron Systems). The FITC (488 nm) channel was used to detect and image the neurons with XCellence pro imaging software and a binning of 4 × 4 (336 × 256). The exposure time was set to 30 ms at a light intensity of 23% to reduce bleaching. For time-lapse imaging 500 frames were acquired at a cycle time of 0.2 s. To examine the baseline activity of the cells, 3 image sequences were recorded at 150 s intervals, corresponding to a total recording time of 10 min. For analysis, regions of interest (ROI) were set around the cell bodies of single neurons using ImageJ. Additionally, a ROI was drawn on an area without cells to perform a background correction. To evaluate Ca^2+^ changes, the amplitude and frequency of fluorescence maxima were determined using a MatLab-based self-developed protocol. The following equation was used to calculate the change in fluorescence intensity: ΔF/F0 = [(F-B)-(F0-B0)] / (F0-B0), where F0 and B0 represent the mean gray level of the selected ROIs under resting conditions identified by the absence of changes in fluorescence intensity. The background correction was done using the smallest standard deviation of the measured fluorescence intensity and the resulting values were used as baseline for the amplitude detection. To compensate for bleaching, the recorded Ca^2+^ traces were processed using a high-pass-filter, which was maintained constant within the experimental series.

### Organotypic Hippocampal Slice Cultures

Organotypic hippocampal cultures were prepared as previously described (Stoppini et al., 1991; Michaelsen-Preusse et al., 2014). Briefly, C57BL/6 BDNF-KO mice of either sex were decapitated on postnatal day 4/5 (P4/5) and the hippocampi were dissected in ice-cold Gey’s Balanced Salt Solution (GBSS) supplemented with glucose, Kynurenic acid and adjusted to pH 7.2. Transverse hippocampal slices were cut using a McIlwain tissue chopper at a thickness of 400 μm. The slices were placed on Millicells© membrane inserts (Millipore) and cultivated at 37°C, 5% CO_2_ and 99% humidity in a medium containing 50% BME (Eagle, with Hanks salts without glutamine), 25% Hank’s Buffered Salt Solution (HBSS), 1% glucose, 25% horse serum (HyClone), and 0.5% L-glutamine. Three days after the preparation a mixture of mitotic inhibitors (uridine, cytosine-β-D-arabinofuranoside*hydrochloride and 5-fluoro-2′-deoxyuridine; 4, 6 × 10^–6^ M each) was applied for 24 h followed by a complete medium exchange. Once a week half of the medium was exchanged.

### Extracellular Field Recordings

Field excitatory postsynaptic potentials (fEPSPs) were recorded in the stratum radiatum of the CA1 region in organotypic hippocampal slices. The slices were placed in a submerged recording chamber and perfused with carbogenated ACSF (32°C) at a rate of ∼1.5 ml/min. The Schaffer collaterals were electrically stimulated with a monopolar tungsten electrode (WPI, United States) at a frequency of 0.1 Hz and fEPSPs were recorded with a borosilicate glass capillary (resistance 1–3 MΩ) filled with 3 M NaCl at a depth of ∼35–70 μm. The stimulation intensity was adjusted to an fEPSP slope of −0.2 mV/ms for measurements across different slices on the same recording day during the 10 min baseline recording. LTP was induced by applying theta burst stimulation (TBS: 10 trains of four pulses at 100 Hz in a 200 ms interval, repeated three times) and recorded for 30 min.

Data of electrophysiological recordings were collected and analyzed with LABVIEW software (National Instruments). The slope of fEPSPs elicited by stimulation of the Schaffer collaterals was measured over time and normalized to the baseline. In this context successful LTP induction was defined as a fEPSP slope larger than 120% of the baseline for the last 5 min after TBS.

### Statistical Analysis

Statistical analysis and graph plotting was performed with GraphPad Prism 6 (GraphPad Software Inc., United States). The data are presented as mean ± SEM. A two-tailed unpaired Student’s *t*-test was used to compare two treatment groups. For the comparison of more than two groups a one-way ANOVA followed by a Bonferroni’s multiple comparisons *post hoc* test was used. The intersections/distance curves of the Sholl analysis and the fEPSP recordings (LTP) were tested using a repeated measures two-way ANOVA and the dendritic spine classification using a two-way ANOVA followed by a Bonferroni’s multiple comparisons *post hoc* test.

## Results

### Application of ZEB85 Affects the Dendritic Architecture of Hippocampal Neurons

#### ZEB85 Treatment Increases Neurite Complexity in Developing Primary Hippocampal Neurons

**FIGURE 1:**
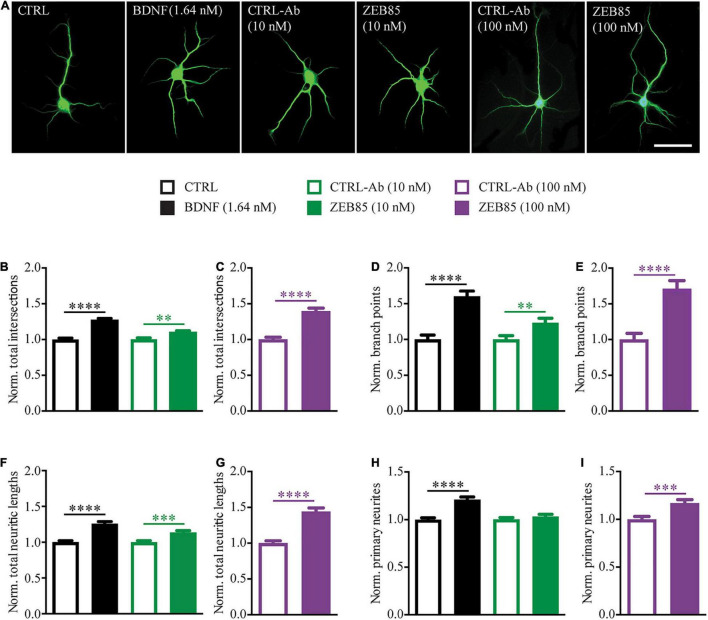
ZEB85 increases neurite complexity of developing hippocampal neurons. Primary hippocampal neurons DIV3 were treated for 4 days with ZEB85 (10 nM and 100 nM), control-antibody (CTRL-Ab) or with BDNF (1.64 nM) and its respective control (CTRL). **(A)** Representative images for the different treatment groups immunostained for MAP2 at DIV7. Scale bar, 50 μm. **(B–I)** Sholl analysis was performed to analyze neurite complexity and data was normalized to the respective controls: **(B,C)** total number of intersections, **(D,E)** number of branch points, **(F,G)** total neurite length and **(H,I)** number of primary neurites. (CTRL, CTRL-Ab 10 nM and ZEB85 10 nM: *n* = 141 neurons; BDNF: *n* = 140; CTRL-Ab 100 nM: *n* = 91; ZEB85 100 nM: *n* = 94; N = 3 independent experiments). The data are presented as mean ± SEM and were tested using an unpaired *t*-test. ^**^p < 0.01; ^***^p < 0.001; ^****^p < 0.0001.

Among the best-established *in vitro* effects of BDNF is its ability to promote neurite outgrowth and complexity in pyramidal neurons (McAllister et al., 1995; Ji et al., 2010; Kellner et al., 2014). Thus, to assess whether treatment with ZEB85 mimics the effects of BDNF on the architecture of developing neurons, low-cell density primary hippocampal cultures were treated starting at DIV3 for 4 days with ZEB85, BDNF or the respective controls (control-antibody and 0.1% BSA). At DIV7 the neurons were fixed, immunostained for MAP2 (Figure 1A), and a Sholl analysis was performed to analyze the neurite complexity of individual neurons (Supplementary Figures 1A,B, 10 nM *F*_(3,559)_ = 25.62 *p* < 0.0001, 100mM *F*_(1,183)_ = 53.88, *p* < 0.0001). As previously shown, BDNF (1.64 nM) treatment significantly increased the number of neurite intersections compared to its control (Figure 1B, CTRL vs. BDNF *p* < 0.0001). Moreover, both the lower (10 nM) and the higher (100 nM) concentration of ZEB85 also led to a significant increase in the total number of intersections compared to the respective concentrations of the control-antibody (Figure 1B, CTRL-Ab 10 nM vs. ZEB85 10 nM *p* < 0.01; Figure 1C, *p* < 0.0001; for the raw data see Supplementary Figure 1C). Also, the number of branch points was significantly increased by the BDNF and ZEB85 treatments (Figure 1D, CTRL vs. BDNF *p* < 0.0001; CTRL-Ab 10 nM vs. ZEB85 10 nM *p* < 0.01; Figure 1E, *p* < 0.0001; for the raw data see Supplementary Figure 1D) compared to the respective controls as well as the total neurite length (Figure 1F, CTRL vs. BDNF *p* < 0.0001; CTRL-Ab 10 nM vs. ZEB85 10 nM *p* < 0.001; Figure 1G, *p* < 0.0001; for the raw data see Supplementary Figure 1E). Finally, while the number of primary neurites was significantly higher in hippocampal neurons treated with BDNF and with 100 nM of ZEB85 (Figure 1H, CTRL vs. BDNF *p* < 0.0001; Figure 1I, *p* < 0.001; for the raw data see Supplementary Figure 1F), the lower 10 nM concentration of ZEB85 had no significant effect, indicating a dose-dependent effect (Figure 1H).

Taken together these results indicate that, ZEB85 TrkB-agonist antibody is able to mimic BDNF action in positively influencing the dendritic architecture of developing neurons.

#### ZEB85 Rescues Dendritic Defects Observed in *Bdnf*-Knockout Parvalbumin-Interneurons

**FIGURE 2:**
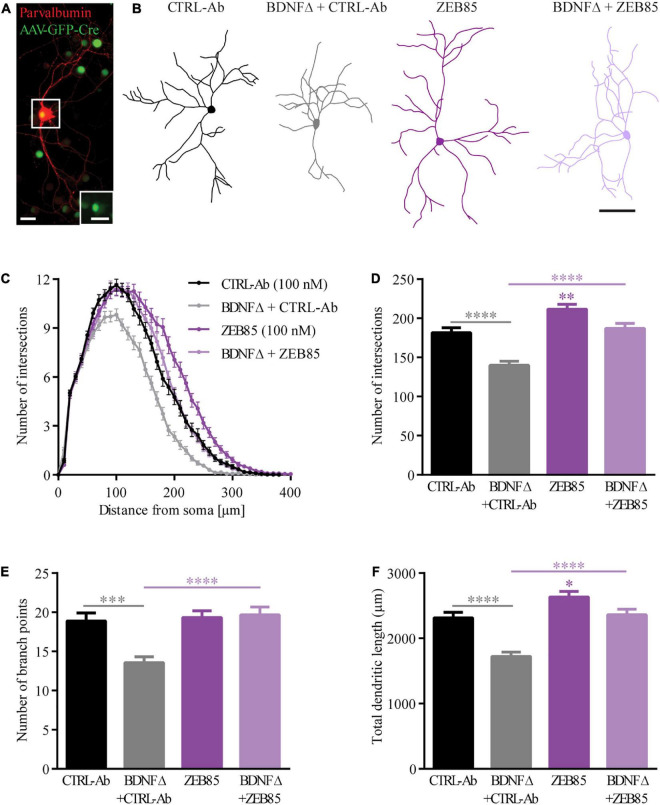
ZEB85 rescues the dendritic complexity of bdnf-KO PV+ interneurons. **(A)** Representative image of an AAV-CRE-GFP transduced parvalbumin-positive (PV^+^) interneuron in primary BDNF^fl/fl^ hippocampal cultures. The inserted image (dotted square) shows the nuclear GFP expression of the transfected PV^+^ interneuron. Both scale bars, 25 μm. **(B)** Neurolucida tracings used for Sholl analysis from PV^+^ interneurons DIV21 treated at DIV0 with ZEB85 (100 nM) or control-antibody (CTRL-Ab) either BDNF-depleted (BDNFΔ) or not. Scale bar, 100 μm. **(C)** Sholl analysis curves, with the number of intersections plotted against the distance from the cell body. Graphs show the **(D)** total number of intersections, **(E)** number of branch points and **(F)** total dendritic length of PV^+^ interneurons. (CTRL-Ab, ZEB85 and BDNFΔ + ZEB85: *n* = 74 neurons; BDNFΔ + CTRL-Ab: *n* = 75; *N* = 3 independent experiments). The data are presented as mean ± SEM and were tested using a **(C)** repeated measures two-way ANOVA and **(D–F)** one-way ANOVA with Bonferroni post-test. *p < 0.05; ^**^p < 0.01; ^***^p < 0.001; ^****^p < 0.0001.

BDNF-deprived GABAergic interneurons in different brain regions show marked alterations in their dendritic architecture both *in vivo* and *in vitro* (Rauskolb et al., 2010; Zagrebelsky et al., 2018; Nieto-Estevez et al., 2021) being considerably smaller and less complex than their relative controls. The altered dendritic architecture of BDNF-deprived GABAergic interneurons in primary hippocampal cultures could be completely rescued by the application of exogenous BDNF during the entire culturing period (Zagrebelsky et al., 2018). Here a possible role of ZEB85 was assessed in rescuing the dendritic complexity of BDNF-deprived parvalbumin-positive (PV^+^) interneurons. Therefore, BDNF depletion was obtained in primary BDNF^fl/fl^ hippocampal neurons transduced (BDNFΔ) with an AVV-CRE-GFP on the day of preparation (DIV0). Transduced and non-transduced cultures were treated with 100 nM of ZEB85 once weekly starting at DIV0 and fixed and stained for parvalbumin at DIV21 (Figure 2A). BDNF-depleted, PV^+^ interneurons treated with the control-antibody (BDNFΔ + CTRL-Ab) were visibly smaller and less complex compared to the non-transduced control-antibody treated neurons (from here on named control; CTRL-Ab; Figure 2B). Further, BDNF-deprived neurons treated with ZEB85 (BDNFΔ + ZEB85) showed a similar size and complexity compared to control neurons (Figure 2B). Quantitative analysis of dendritic complexity using the Sholl analysis confirmed this impression. BDNF-depleted PV^+^ interneurons treated with the control-antibody revealed a significant reduction in the number of intersections especially for the mid-distal part of the dendritic tree compared to the control neurons (Figure 2C, *F*_(3,284)_ = 22.09, *p* < 0.0001). This result was also reflected in a significant reduction in the total number of intersections as well as the number of branch points, which were reduced by ca. 30% (Figure 2D, *F*_(3, 293)_ = 24.6, CTRL-Ab vs. BDNFΔ + CTRL-Ab *p* < 0.0001) and ca. 40% (Figure 2E, *F*_(3, 293)_ = 10.1, *p* < 0.001), respectively. Consistent with being less complex, BDNF-deprived PV^+^ interneurons were also significantly shorter (Figure 2F, *F*_(3, 294)_ = 22.6, *p* < 0.0001). The markedly reduced complexity and size of BDNF-deprived PV^+^ interneurons could be completely rescued by treatment with 100 nM of ZEB85 (BDNFΔ + CTRL-Ab vs. BDNFΔ + ZEB85 Figure 2D, *p* < 0.0001; Figure 2E, *p* < 0.0001; Figure 2F, *p* < 0.0001). Moreover, treatment with ZEB85 in non-transduced neurons also increased the number of intersections for the more distal part of the dendritic tree (Figure 2C) and significantly increased the total number of intersections by 16% (Figure 2D, CTRL-Ab vs. ZEB85 *p* < 0.01) and the total dendritic length by 14% (Figure 2F, *p* < 0.0001) compared to the control.

Taken together these results show a clear and reproducible effect of the novel TrkB-agonist antibody ZEB85 in the maintenance of the dendritic architecture by promoting dendritic branching in BDNF-depleted GABAergic interneurons.

### ZEB85 Treatment Affects Dendritic Spine Density in Mature Hippocampal Neurons

#### Treatment With ZEB85 Affects Spine Type Morphology in Mature Primary Hippocampal Neurons

**FIGURE 3:**
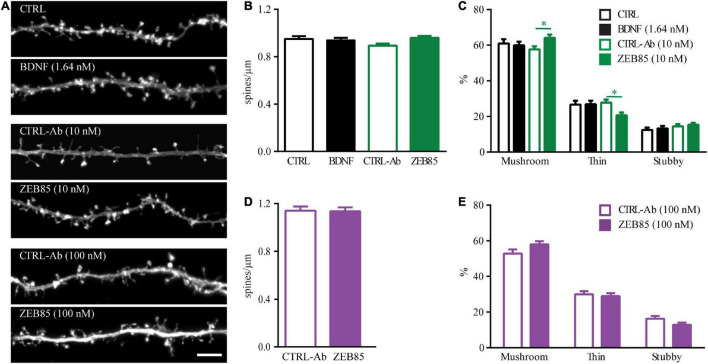
ZEB85 affects spine type distribution in mature primary hippocampal neurons. **(A)** Representative dendritic segments with dendritic spines from feGFP transfected hippocampal neurons treated for 24 h with ZEB85 (10 nM and 100 nM), BDNF and the corresponding controls. Scale bar, 5 μm. **(B,D)** Quantification of spine density (CTRL: *n* = 58 dendrites/neurons; BDNF: *n* = 59; CTRL-Ab 10 nM: *n* = 53; ZEB85 10 nM: *n* = 58; CTRL-Ab 100 nM: *n* = 45; ZEB85 100 nM: *n* = 44; *N* = 4 independent experiments). The data are presented as mean ± SEM and were tested using **(B)** a one-way ANOVA with Bonferroni post-test or **(D)** an unpaired *t*-test. **(C,E)** Quantification of spine type distribution (CTRL: *n* = 40; BDNF: *n* = 37; CTRL-Ab 10 nM: *n* = 52; ZEB85 10 nM: *n* = 52; CTRL-Ab 100 nM: *n* = 28; ZEB85 100 nM: *n* = 27; *N* = 3 independent experiments). The data are presented as mean ± SEM and were tested using a two-way ANOVA with Bonferroni post-test. *p < 0.05.

While some studies have described an increase in dendritic spine density and differences in spine morphology upon treatment with exogenous BDNF for hippocampal pyramidal neurons in primary cultures (Ji et al., 2005, 2010) or organotypic slice cultures (Tyler and Pozzo-Miller, 2001), other studies could not reproduce these effects and found no alterations in dendritic spines upon application of BDNF (Kellner et al., 2014). Thus, we started by testing, under our culture conditions whether BDNF shows an effect on dendritic spine number and morphology and then whether the effect of the TrkB agonist antibody ZEB85, differs from that of BDNF. Therefore, mature primary hippocampal cultures were transfected with feGFP and treated with BDNF, both concentrations of ZEB85 (10 nM and 100 nM) and with the respective control for 24 h. As previously shown (Kellner et al., 2014) BDNF treatment showed no effect on the number of dendritic spines (Figures 3A,B). Similarly, treatment with both concentrations of ZEB85 showed no changes in dendritic spine density relative to the controls (Figures 3B,D). Next, spine type distribution was analyzed. Therefore, dendritic spines were classified based on their length, head and neck width into mushroom, thin or stubby spines. Both BDNF and 100 nM of ZEB85 had no effect on the spine type distribution (Figures 3C,E). However, interestingly, treatment with the lower concentration of ZEB85, 10 nM, leads to a slightly, albeit significantly higher proportion of mushroom- and a lower one for thin-type spines (Figure 3C, F*_*Interaction*_*_(6, 531)_ = 3.6, both *p* < 0.05) supporting a role of BDNF/TrkB signaling in regulating spine type distribution as previously shown (Rauskolb et al., 2010).

#### ZEB85 Rescues Amyloid Beta_1–42_-Induced Spine Loss

**FIGURE 4:**
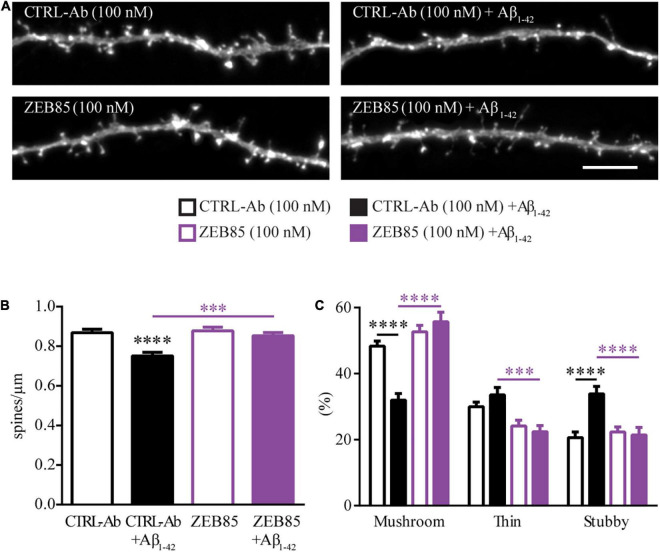
ZEB85 rescues Amyloid beta1-42-induced spine loss. **(A)** Representative dendritic segments with dendritic spines from feGFP transfected hippocampal neurons either pretreated with ZEB85 (100 nM) or control-antibody (CTRL-Ab) for 15 min, followed by a 6 h treatment with amyloid-β1-42 or left further untreated. Scale bar, 5 μm. **(B)** Quantification of spine density (CTRL-Ab and ZEB85: *n* = 47 dendrites/neurons; CTRL-Ab + Aβ: *n* = 49; ZEB85 + Aβ: *n* = 48). *N* = 3 independent experiments. The data are presented as mean ± SEM and were tested using a one-way ANOVA with Bonferroni post-test. **(C)** Quantification of spine type distribution (*n* = 30; *N* = 3 independent experiments). The data are presented as mean ± SEM and were tested using a two-way ANOVA with Bonferroni post-test. ^***^p < 0.001; ^****^p < 0.0001.

The loss of dendritic spines and the correlated loss of synapses is an early pathological alteration observed in Alzheimer’s disease (AD), and is directly correlated to memory loss and cognitive decline (Dorostkar et al., 2015). Dendritic spine pathology in AD has been associated with the effect of soluble Amyloid beta_1–42_ (Aβ_1–42_) oligomers (Brouillette, 2014; Dorostkar et al., 2015; Kommaddi et al., 2018; Patnaik et al., 2020). Further, the protein and mRNA levels for BDNF and TrkB are significantly decreased in AD (Ferrer et al., 1999; Ginsberg et al., 2010), and previous studies have shown that treatment with BDNF restored spine abnormalities in AD mouse models *in vivo* (de Pins et al., 2019) and *in vitro* (Meng et al., 2013). To test whether ZEB85 is able to restore the spine pathology observed upon application of soluble Aβ_1–42_ oligomers (Patnaik et al., 2020), feGFP expressing primary hippocampal neurons (DIV21) were pre-treated with 100 nM ZEB85 or the control-antibody for 15 min and then co-treated with oligomeric Aβ_1–42_ (oAβ; 500 nM) or the respective control (DMSO in PBS) for 6 h. Neurons co-treated with oAβ and control-antibody displayed a visibly reduced dendritic spine number (Figure 4A) compared to control neurons treated only with control-antibody. The oAβ-induced decrease in dendritic spine density was completely prevented by a co-treatment with ZEB85. Indeed, the quantification confirmed that oAβ plus control-antibody treated neurons had a significantly reduced spine density compared to control neurons (Figure 4B, *F*_(3, 187)_ = 10.9, CTRL-Ab + Aβ vs. CTRL-Ab, *p* < 0.0001) and that this loss of dendritic spines was absent in neurons co-treated with ZEB85 showing a spine density equivalent to the one of control neurons (Figure 4B, CTRL-Ab + Aβ vs. ZEB85 + Aβ *p* < 0.001). In line with the previous spine density data (Figures 3B,D), a 6 h treatment with ZEB85 had no effect on spine density of healthy neurons compared to control neurons (Figure 4B). Further, treatment with Aβ_1–42_ also lead to changes in the spine type distribution (Figure 4C, F*_*Interaction*_*_(6, 348)_ = 22.3, *p* < 0.0001). The proportion of mushroom type spines was significantly reduced (CTRL-Ab + Aβ vs. CTRL-Ab *p* < 0.0001), while the proportion of stubby spines increased (*p* < 0.0001). The oAβ-induced shift in spine type distribution could be prevented with the co-treatment of ZEB85. Indeed, the proportion of mushroom spines was significantly increased (CTRL-Ab + Aβ vs. ZEB85 + Aβ *p* < 0.0001) and the one of the stubby (*p* < 0.001) and thin (*p* < 0.0001) spines was reduced to control level.

These results indicate the ability of the ZEB85 TrkB-agonist antibody to preserve the dendritic spine density and morphology of hippocampal neurons exposed to amyloid beta.

### ZEB85 in Regulating TrkB Signaling and Neuronal Activity

#### ZEB85 Affects Long-Term Potentiation

**FIGURE 5:**
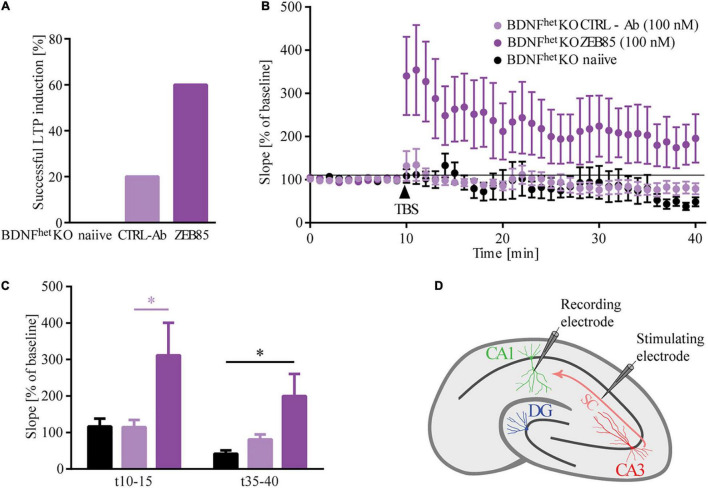
ZEB85 rescues LTP deficits in hippocampal slices of BDNF knockout mice. fEPSP were recorded at the CA3-CA1 pathway of DIV21/22 organotypic hippocampal slice cultures prepared from heterozygous BDNF-knockout mice treated for 5–6 days with 100 nM ZEB85 or CTRL-Ab or left untreated. **(A)** Graph shows the percentage of successful LTP inductions. The criterion for a successful LTP induction was based on the fact that fEPSPs for the last 5 min after TBS were larger than 120% of the baseline. **(B)** fEPSP recordings over time. Baseline was recorded for 10 min after which TBS was applied and LTP was measured for another 30 min. The data are presented as mean ± SEM and were tested using a repeated measure two-way ANOVA. **(C)** Graph shows the mean values of the first and last 5 min after LTP induction. The data are presented as mean ± SEM and were tested using a one-way ANOVA with Bonferroni post-test. **(D)** Schematic of the fEPSP recordings in a hippocampal slice with electrode placement in the CA3-CA1 pathway. (Naïve: *N* = 5 mice, *n* = 8 slices; CTRL-Ab and ZEB85: *N* = 6 mice, *n* = 10 slices). *p < 0.05.

BDNF signaling *via* TrkB has been previously shown to be crucial for the induction and maintenance of long-term potentiation (LTP; Korte et al., 1995; Minichiello, 2009; Zagrebelsky and Korte, 2014). Therefore, we next investigated whether activation of the TrkB receptor *via* the application of ZEB85 may rescue the LTP defects observed in organotypic hippocampal slice cultures of heterozygous BDNF knockout mice (BDNF^het^KO). The slice cultures were treated starting at DIV16 for 5−6 days with 100 nM of ZEB85, after which field excitatory postsynaptic potential (fEPSP) recordings were performed. Therefore, the Schaffer collaterals were stimulated and fEPSPs were recorded in the CA1 region (Figure 5D). All recordings with a stable baseline were included, comprising those that showed no induction of LTP or those where the fEPSP slope decreased after TBS. The results show that, while LTP could only be induced in 20% of BDNF^het^KO hippocampal slice cultures treated with control-antibody, 60% of BDNF^het^KO hippocampal slice cultures treated with ZEB85 showed successful LTP induction (Figure 5A). In BDNF^het^KO hippocampal slice cultures treated with control-antibody TBS resulted in a weak and short-lasting potentiation after which the fEPSP slope decreased slightly below the baseline (Figure 5B, *F*_(2, 25)_ = 3.59 *p* < 0.05). In contrast, ZEB85 treated BDNF^het^KO hippocampal slice cultures displayed a robust and lasting potentiation after TBS (Figure 5B, *F*_(2, 25)_ = 3.6, *p* < 0.05). Both the first 5 mins and also the last 5 mins after TBS showed an increase in the fEPSP slope which was significant for the first 5 mins relative to the baseline compared to the treatment with control antibodies (Figure 5C, t10−15: *F*_(2, 25)_ = 4.0, *p* < 0.05; t35−40: *F*_(2, 25)_ = 4.9 *p* < 0.05). Of note, treatment with the control-antibody had no marked impact on LTP compared to naïve, untreated slices.

#### ZEB85 Application Leads to Activation of TrkB and Its Downstream Signaling Cascade and Increases Neuronal Activity

**FIGURE 6:**
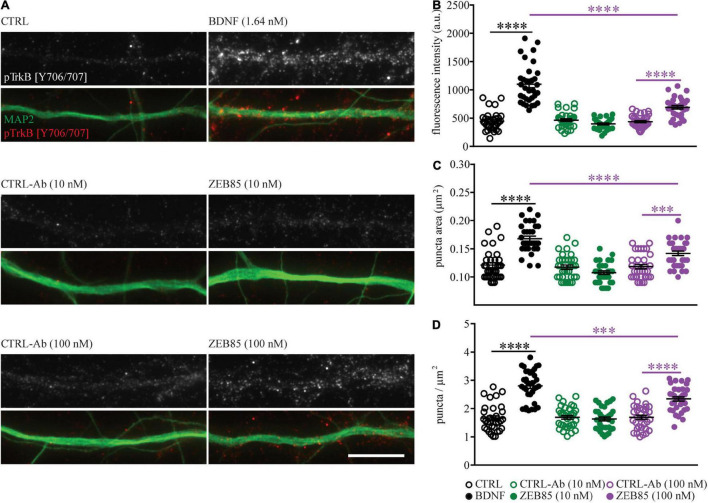
ZEB85 leads to TrkB phosphorylation. Primary hippocampal cultures DIV21 were treated for 15 min with ZEB85 (10 nM and 100 nM), BDNF (1.64 nM) and the corresponding controls. **(A)** Representative dendritic segments immunostained for MAP2 and the TrkB phosphorylation site Tyr706/707 (pTrkB) shown for the different treatment groups. Scale bar, 10 μm. **(B)** Quantification of pTrkB fluorescence intensity, **(C)** pTrkB puncta area, and **(D)** pTrkB density. (*n* = 33 dendrites/neurons; *N* = 3 independent experiments). The data are presented as mean ± SEM and were tested using a one-way ANOVA with Bonferroni post-test. ^***^p < 0.001; ^****^p < 0.0001.

Merkouris et al. (2018) showed that ZEB85 leads to TrkB phosphorylation at site Tyr706/707 in human neurons derived from hES cells at a concentration of 5 nM and also in cultured mouse cortical neurons at a concentration of 50 nM. We used immunocytochemistry to determine if the lower concentrations of ZEB85 used for the current experiments would also lead to TrkB activation in primary mouse hippocampal neurons. Therefore, DIV21 primary hippocampal cultures (plated at a cell density of 3.5 × 10^4^/cm^2^) were treated with 10 nM and 100 nM of ZEB85 and the respective concentrations of control-antibody or BDNF. After a 15 min treatment the cultures were immunostained for MAP2 and for the phosphorylated TrkB site Tyr706/707 (pTyr706/707). Treatment with BDNF as well as with 100 nM of ZEB85 visibly resulted in a stronger fluorescent intensity for the pTyr706/707 staining compared to the respective controls (Figure 6A). Quantification confirmed that both, BDNF and 100 nM of ZEB85 significantly increased the levels of TrkB phosphorylation (pTrkB) at individual sites, as shown by the higher pTrkB puncta fluorescent intensity (Figure 6B, *F*_(5, 192)_ = 68.2, CTRL vs. BDNF *p* < 0.0001; CTRL-Ab 100 nM vs. ZEB85 100 nM *p* < 0.0001) the bigger pTrkB puncta area than under control conditions (Figure 6C, *F*_(5, 192)_ = 30.5, CTRL vs. BDNF *p* < 0.0001; CTRL-Ab 100 nM vs. ZEB85 100 nM *p* < 0.001) and the number of sites with activated TrkB shown by the greater pTrkB puncta density (Figure 6D, *F*_(5, 192)_ = 39.5, CTRL vs. BDNF *p* < 0.0001; CTRL-Ab 100 nM vs. ZEB85 100 nM *p* < 0.0001). BDNF treatment led to a significantly stronger TrkB activation compared to 100 nM of ZEB85 (Figure 6B *p* < 0.0001, Figure 6C *p* < 0.0001, and Figure 6D *p* < 0.001). The lower concentration of 10 nM of ZEB85 did not result in any detectable phosphorylation of TrkB Tyr706/707.

**FIGURE 7:**
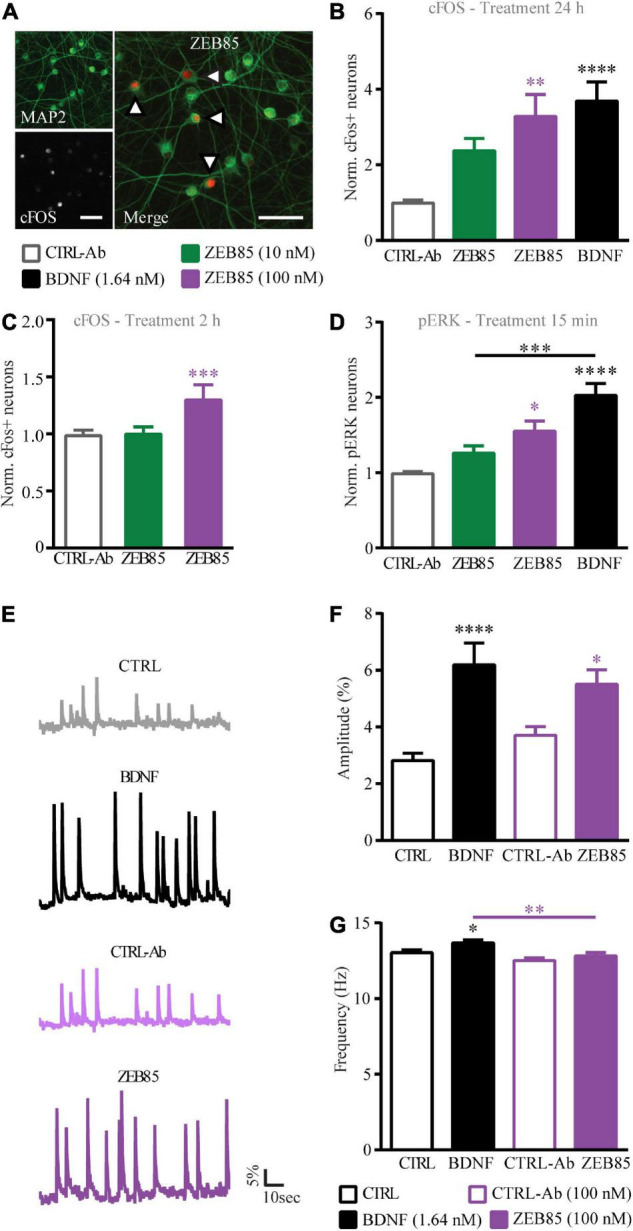
ZEB85 induces cFOS and pERK expression and promotes intracellular calcium dynamics. **(A)** Primary hippocampal cultures DIV21 were immunostained for MAP2 (left insert above) and cFos (left insert below) or pERK (not shown). The arrows in the merged image (right) indicate cFOS expressing MAP2 positive neurons. Scale bar, 50 μm. The number of cFOS/pERK positive neurons was calculated over total MAP2 positive neurons with a defined ROI, and is normalized to the controls **(B–D)**. Quantification of cFOS positive neurons treated for panels **(B)** 24 h (CTRL-Ab: *n* = 19 coverslips; BDNF: *n* = 20; ZEB85 10 nM: *n* = 9; ZEB85 100 nM: *n* = 11 coverslips; for 10 nM *N* = 4 independent experiments and for 100 nM; *N* = 3 independent experiments) or **(C)** 2 h (CTRL-Ab: *n* = 46; ZEB85 10 nM: *n* = 23; ZEB85 100 nM: *n* = 24; *N* = 8 independent experiments) and quantification of panel **(D)** pERK positive neurons treated for 15 min (CTRL-Ab and BDNF: *n* = 15; ZEB85 10 nM: *n* = 8; ZEB85 100 nM: *n* = 6; *N* = 3 independent experiments). Neurons were treated with ZEB85 (10 nM and 100 nM) and control-antibody (CTRL-Ab) and for panels **(B,D)** additionally with BDNF. **(E)** Ca^2+^-imaging traces of OGB loaded neurons after a 24 h treatment with BDNF, 100 nM ZEB85 and the respective controls. The percentage change in fluorescence intensity over time is illustrated. Scale bars are 5% vertical and 10 sec horizontal. **(F)** Ca^2+^ transient amplitude and **(G)** frequency of the different treatment groups. (CTRL: *n* = 97 neurons; BDNF: *n* = 95; CTRL-Ab *n* = 78; ZEB85: *n* = 85; *N* = 3 independent experiments). The data are presented as mean ± SEM and were tested using a one-way ANOVA with Bonferroni post-test. *p < 0.05; ^**^p < 0.01; ^***^p < 0.001; ^****^p < 0.0001.

Next, the activation of the downstream intracellular signal transduction pathway of TrkB was investigated upon ZEB85 application, specifically analyzing the cFOS and pERK expression levels. Thus, primary hippocampal cultures DIV21 were treated with 10 nM and 100 nM of ZEB85 or the control-antibody and the analysis was performed by counting cFOS or pERK positive nuclei of MAP2 positive neurons (Figure 7A). The activity-dependent immediate early gene cFOS is upregulated upon BDNF-mediated TrkB signaling (Marty et al., 1996; Pizzorusso et al., 2000; Gascon et al., 2005; Ohba et al., 2005) and was therefore used to detect activation downstream of TrkB. A 24 h treatment with BDNF as well as treatment with 100 nM of ZEB85 resulted in a significant increase in the number of cFOS expressing neurons compared to the respective control (Figure 7B, *F*_(3, 55)_ = 10.5 CTRL-Ab vs. ZEB85 100 nM *p* < 0.01 and vs. BDNF, *p* < 0.0001). Also, a shorter treatment of 2 h led to a significant increase in cFOS expression with 100 nM of ZEB85 (Figure 7C, *F*_(2, 90)_ = 7.6, CTRL-Ab vs. ZEB85 100 nM *p* < 0.001). While the lower concentration of 10 nM of ZEB85 led to an increase in cFOS expression, this was not significant. Further, a treatment of 15 min also only led to a significant increase in pERK positive neurons (Supplementary Figure 2) with both BDNF and 100 nM of ZEB85 (Figure 7D, *F*_(3, 40)_ = 17.6, CTRL-Ab vs. ZEB85 100 nM *p* < 0.05 and vs. BDNF *p* < 0.0001) and not with 10 nM of ZEB85 (Figure 7D). The effect on cFOS and pERK expression was smaller for ZEB85 compared to BDNF, with a significant difference for the number of pERK positive neurons (Figure 7D, *p* < 0.001). Based on the above results, showing that ZEB85 increases the expression of the activity-related proteins cFOS and pERK and previous studies reporting that BDNF/TrkB signaling evokes calcium transients in hippocampal neurons (Lang et al., 2007), we hypothesized that, similar to BDNF, ZEB85 might modulate neuronal activity. Thus, the basal spontaneous neuronal activity in OGB loaded primary hippocampal neurons after a 24 h treatment with 100 nM of ZEB85 or BDNF, as a positive control was compared to the one of vehicle treated neurons by measuring the changes in fluorescence intensity within the neuronal cell bodies (Figure 7E). Indeed, both BDNF and ZEB85 significantly increased the amplitude of Ca^2+^ signals (Figure 7F, *F*_(3, 356)_ = 9.8, CTRL vs. BDNF *p* < 0.0001; CTRL-Ab vs. ZEB85 *p* < 0.05). However, only BDNF increased the frequency significantly (Figure 7G, *F*_(3, 341)_ = 6.1, CTRL vs. BDNF *p* < 0.05).

Taken together the results above show that application of the ZEB85 TrkB-agonist antibody induces TrkB phosphorylation and its downstream signaling and increases the strength of neuronal activity at concentrations at which an effect can be seen on the dendritic architecture of developing pyramidal neurons, of GABAergic interneurons and on activity-dependent synaptic plasticity in the hippocampus.

## Discussion

This study provides an in-depth characterization of the ability of ZEB85, a novel fully human agonist antibody to the TrkB receptor, to mimic the effects of the native ligand BDNF. In a series of well-established assays, ZEB85 reproduces effects similar to those elicited by BDNF in modulating the architecture and function of developing and mature mouse hippocampal neurons, as follows:

(1) ZEB85 promotes neurite growth in developing pyramidal hippocampal neurons and rescues dendritic complexity in BDNF-deficient hippocampal interneurons;

(2) ZEB85 rescues the impairment in long-term potentiation (LTP) in BDNF heterozygous knockout mice-derived hippocampal slice cultures;

(3) treatment of hippocampal primary neurons with ZEB85 results in increased expression of the immediate early gene cFOS and the TrkB downstream activated kinase pERK, two immediate early genes (IEGs) downstream of the BDNF/TrkB signaling and stimulates TrkB phosphorylation;

(4) ZEB85 treatment increases the strength of neuronal activity;

(5) co-treatment of primary hippocampal neurons with amyloid beta_1–42_ (Aβ_1–42_) oligomers and ZEB85 prevents the Aβ-induced loss of dendritic spines.

These results strongly support a specific action of ZEB85 in modulating the structure and function of mouse hippocampal neurons by specifically activating the signaling *via* the TrkB receptor opening the possibility of using TrkB agonist antibodies both as therapeutic agents and as research tools to study TrkB-dependent signal transduction and its biological consequences from cell culture to *in vivo* approaches.

BDNF signaling *via* its receptor TrkB positively modulates the dendritic architecture of developing hippocampal neurons. Indeed, BDNF and TrkB are expressed by developing hippocampal pyramidal neurons *in vivo* and *in vitro* (Swanwick et al., 2004; Luikart et al., 2005), and exogenous application of BDNF promotes branching and elongation of developing neurites (Alonso et al., 2004; Ji et al., 2005, 2010; Kellner et al., 2014). While an acute, short activation of TrkB results in longer neurites, a prolonged TrkB activation, by gradually increasing BDNF promotes neurite branching and more complex dendritic trees (Ji et al., 2010). The current results show that treatment with the TrkB agonist antibody ZEB85 reproduces both these actions exerted by exogenous BDNF on neurite development; ZEB85 application results in longer and more complex dendritic trees possibly in a concentration-dependent manner. Although the treatments were performed in separate experiments, 10 nM ZEB85 reproduces the effects of 1.64 nM BDNF, albeit to a lower degree while 100 nM shows a stronger effect, comparable to that of BDNF on neurite elongation, branching and in promoting the formation of primary neurites. Previously, the effect of ZEB85 on dendritic growth was tested on mouse retinal ganglion cells (Merkouris et al., 2018). There, the effects of 50 nM ZEB85 antibody were roughly equivalent to those of approximately 5 nM BDNF. The higher concentrations used compared to the present study may be explained by the lower penetration of proteins through retinal explants than in dissociated primary hippocampal cultures. While the overall concentration-dependent effects of ZEB85 in the above experiments trend very similar to those observed here and previously with BDNF, the magnitude of ZEB85 effects were generally smaller than for the saturating concentration of BDNF used. BDNF is among the most highly conserved protein ligands known, and as such human BDNF is equipotent in activating human, mouse and rat TrkB. TrkB sequences across species, however, are not as highly conserved possibly explaining why a TrkB agonist antibody derived from a human phage-display library is less potent in activating TrkB on mouse nerve cells. From the present experiments, ZEB85 would appear to be 0.5−1 log less potent on mouse vs. human neurons.

While in some studies the exogenous application of BDNF increases dendritic spine density in mature neurons in primary (Ji et al., 2005, 2010) and organotypic hippocampal cultures (Tyler and Pozzo-Miller, 2001, 2003), others could not reproduce these observations (Kellner et al., 2014). These discrepancies could be explained by differences in cell culture conditions (Danzer and McNamara, 2004; Chapleau et al., 2008), especially regarding the levels of neuronal activity and possibly influencing the amount of endogenous BDNF and thereby a saturating effect on its signaling (Kellner et al., 2014). In this study the effects of exogenous BDNF and ZEB85 were compared strictly under constant conditions and reproduce our previous observations confirming the inability of exogenous BDNF to affect dendritic spine density and spine type distribution (Kellner et al., 2014; Zagrebelsky et al., 2018). While treatment of mature hippocampal neurons with ZEB85 does not affect dendritic spine density, it results in a slight increase in the proportion of mushroom and a decrease in the one of thin spines. This is of interest since it may depend on an action of ZEB85 specifically on the TrkB receptor vs. a mixed action of BDNF both on TrkB and p75^NTR^. Indeed, while p75^NTR^ preferentially mediates the actions of proBDNF, it also binds mature BDNF (Lee and Chao, 2001). BDNF and proBDNF exert opposite actions on dendritic spines: treatment of mature hippocampal neurons with BDNF increases their dendritic spine density, application of proBDNF significantly reduces it (Koshimizu et al., 2009). Furthermore, pyramidal neurons in hippocampal slices cultivated with or without serum show characteristic distributions of dendritic spine types associated with different p75^NTR^-to-TrkB expression levels (Chapleau et al., 2008) supporting the notion that application of ZEB85 might specifically mimic the effects of the BDNF/TrkB ligand-receptor interaction. Indeed, ZEB85 does not recognize p75^NTR^, TrkA or TrkC (Merkouris et al., 2018) suggesting that its effects are indeed exclusively through TrkB.

Application of sublethal concentrations of Aβ_1–42_ oligomers to primary hippocampal neurons is an established *in vitro* model for the synaptic loss typical of the early phases of Alzheimer disease (AD) (Lacor et al., 2007; Jackson et al., 2019). We recently showed an Aβ_1–42_ oligomer-induced dendritic spine loss in primary hippocampal neurons is mediated by p75^NTR^ (Patnaik et al., 2020). Co-treatment with ZEB85 completely prevents the dendritic spine loss upon Aβ_1–42_ oligomer treatment. Whether this is due to the ability of ZEB85 to generically restore the impaired TrkB signaling and thereby promote dendritic spine formation or to a specific competition between the ZEB85-induced TrkB activation and the Aβ_1–42_-p75^NTR^ signaling remains an interesting open question. However, the rescue effect of ZEB85 is highly relevant from a potential therapeutic prospective, particularly with respect to the fact that BDNF protein and mRNA levels are reduced in AD patients (Hock et al., 2000), and that BDNF protects neurons against Aβ toxicity and prevents dendritic spine abnormalities in AD mouse models both *in vivo* (de Pins et al., 2019) and *in vitro* (Meng et al., 2013). In addition to neurodegenerative diseases, alterations in BDNF and TrkB levels have been implicated in psychiatric diseases such as depression (Castrén and Monteggia, 2021) and the action of many antidepressant drugs have been linked to the regulation of TrkB signaling (Casarotto et al., 2021) suggesting that direct activation of TrkB receptors could be a new approach to treat major depression in humans.

*In vivo* and *in vitro* deletion of BDNF results in smaller dendritic trees for GABAergic interneurons (Rauskolb et al., 2010; Zagrebelsky et al., 2018). Moreover, the development of GABAergic neurons has been shown to be regulated by excitatory input through the release of BDNF in an activity-dependent manner (Kohara et al., 2007; Turrigiano, 2007; Hong et al., 2008) and a much larger growth response to exogenous BDNF has been reported for hippocampal GABAergic interneurons than pyramidal neurons (Vicario-Abejon et al., 1998; Rauskolb et al., 2010). We have previously shown that exogenous BDNF application not only rescues the impaired dendritic tree of BDNF-deficient hippocampal GABAergic neurons but it also increases dendritic complexity in the wildtype (Zagrebelsky et al., 2018). Notably, in the current study, application of ZEB85 completely reproduces this pattern for mouse-derived parvalbumin-positive hippocampal interneurons.

The binding of BDNF to TrkB stimulates trans-phosphorylation of multiple Tyr residues, in its intracellular domain (Huang and Reichardt, 2003; Minichiello, 2009). In this study, a treatment with ZEB85 reproduces the effects of BDNF on TrkB phosphorylation at Tyr 706/707, albeit with a significantly lower efficacy, confirming the previous observations of Merkouris et al. (2018) in mouse cortical neurons. Application of ZEB85 to human embryonic stem cell-derived neurons resulted in transcriptional profile changes highly comparable to those of BDNF (Merkouris et al., 2018) suggesting that small but significant differences in rodent vs. human TrkB ectodomain specifically influence the potency of the TrkB agonist antibody on TrkB, without affecting the signaling of the natural ligand BDNF. However, an alternative possibility is that the differences in the effects of BDNF and ZEB85 might derive from a distinct stability of the two molecules. While in our experiments we cannot completely exclude that part of the increased phosphorylation we see is related to TrkA, the previously shown lack of binding of ZEB85 to TrkA (Merkouris et al., 2018) suggests that its application to primary hippocampal neurons specifically activates TrkB. In this context, combining the ZEB85 antibody treatment with a specific loss-of-function approach for TrkB would be of extreme interest as an additional proof of its specificity.

BDNF/TrkB signaling affects neuronal calcium responses and strengthens basal excitatory synaptic transmission (Berninger et al., 1993; Kang and Schuman, 1995, 2000; Lang et al., 2007; Ji et al., 2010). Our results show that BDNF increases the amplitude of neuronal calcium transients, as does ZEB85. The increase in neuronal activity is associated to a significant increase in cFOS expression which is especially strong after a 24 h treatment. Whether the increase in cFOS we observe is a direct consequence of the TrkB activation or it is due to the general increase in neuronal activity cannot be concluded from the present data. Indeed, we previously have shown that the effects of BDNF on neuronal architecture are dependent on neuronal activity (Kellner et al., 2014) possibly indicating an interaction between the two mechanisms. Moreover, BDNF/TrkB signaling has been shown to promote the induction and maintenance of LTP (Akaneya et al., 1997; Ji et al., 2010). Impaired maintenance of LTP in the hippocampus is a typical sign of diminished functional plasticity in mice lacking one or both *bdnf* alleles (Korte et al., 1995) and is rescued upon either virally mediated BDNF re-expression (Korte et al., 1996) or application of recombinant BDNF (Patterson et al., 1996). Similarly, the current results show upon application of ZEB85 a significant rescue of the LTP induction deficit observed in heterozygous BDNF knockout hippocampal slices.

Taken together the results demonstrate that the fully human TrkB agonist antibody ZEB85 elicits comparable responses to BDNF in several assays addressing the BDNF-dependent regulation of neuronal architecture and synaptic plasticity in the mouse hippocampus.

## Data Availability Statement

The raw data supporting the conclusions of this article will be made available by the authors, without undue reservation.

## Ethics Statement

The animal study was reviewed and approved by the TU Braunschweig and the LAVES (Oldenburg, Germany, Az. §4 (02.05) TSchB TU BS).

## Author Contributions

CT, MK, and MZ designed the research. CT and KM performed the research, wrote the manuscript, and analyzed the data. PD and RL contributed to the reagents. CT, PD, RL, MK, and MZ edited the manuscript. All authors contributed to the article and approved the submitted version.

## Conflict of Interest

PD and RL hold stock options with Zebra Biologics, Inc. The remaining authors declare that the research was conducted in the absence of any commercial or financial relationships that could be construed as a potential conflict of interest.

## Publisher’s Note

All claims expressed in this article are solely those of the authors and do not necessarily represent those of their affiliated organizations, or those of the publisher, the editors and the reviewers. Any product that may be evaluated in this article, or claim that may be made by its manufacturer, is not guaranteed or endorsed by the publisher.
