# Supplementary material for: Actions of the TrkB Agonist Antibody ZEB85 in Regulating the Architecture and Synaptic Plasticity in Hippocampal Neurons
Source: Front Mol Neurosci. 2022 Jun 30;15:945348. doi: 10.3389/fnmol.2022.945348 (PMC9280622; doi:10.3389/fnmol.2022.945348)
Supplement: Supplementary file 1 [file Image_1.pdf]

Tacke et al., Supplemental Figure 1

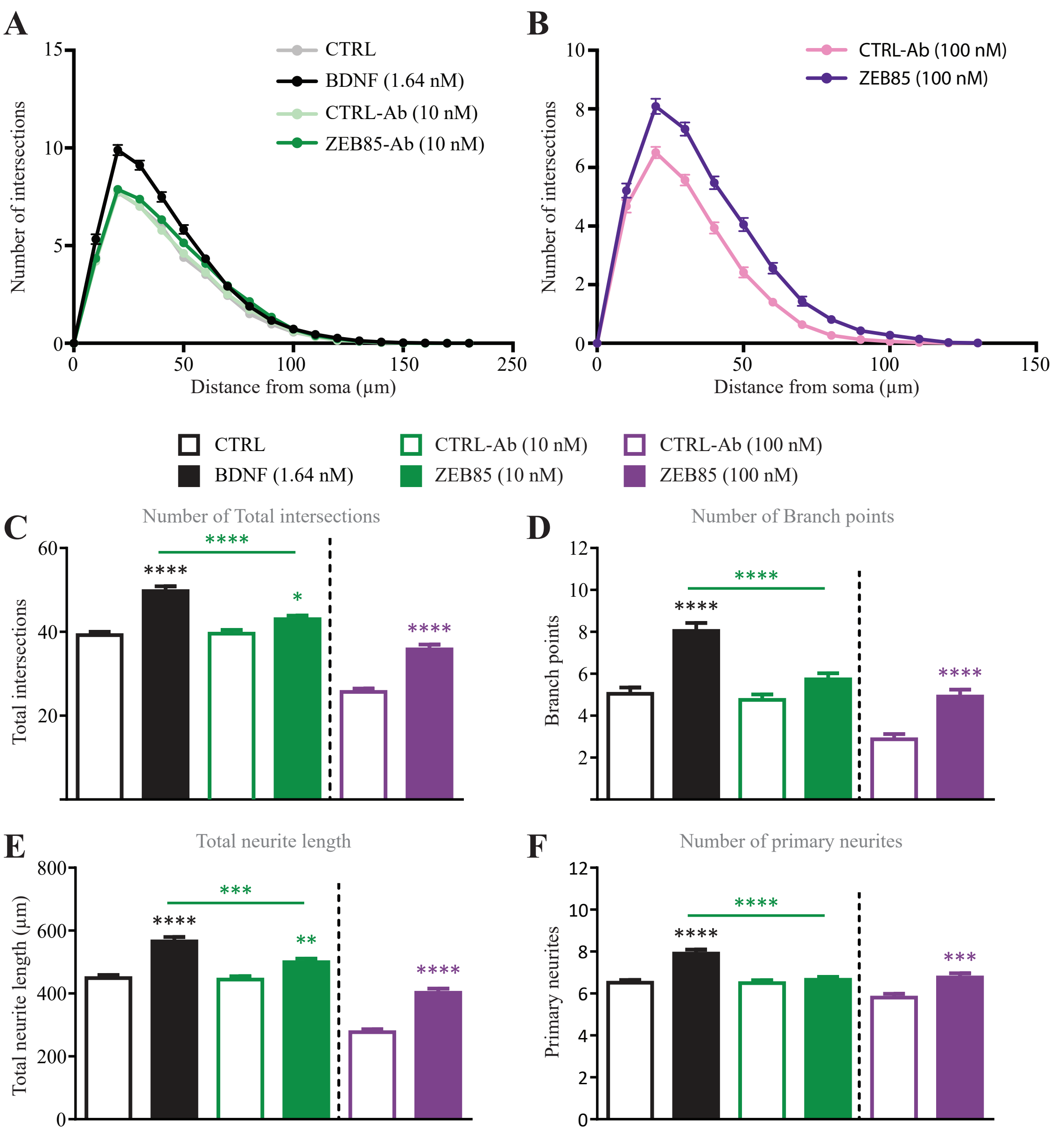

Figure 1 Supplementray: ZEB85 increases neurite complexity of developing hippocampal neurons.

Primary hippocampal neurons were treated for 4 days starting at DIV3 with ZEB85 (10 nM and 100 nM), control-antibody (CTRL-Ab, 10 nM and 100 nM) or with BDNF (1.64 nM) and its respective control, BSA (CTRL). (A & B) Sholl analysis curves, with the number of intersections plotted against the distance from the cell body. Graphs show the (C) total number of intersections, (D) number of branch points, (E) total dendritic length and (F) primary neurites. (CTRL, CTRL-Ab 10 nM and ZEB85 10 nM: n=141 neurons; BDNF: n=140; for (A & B) repeated measures two-way ANOVA and for (C-F) one-way ANOVA plus Bonferroni post-test; CTRL-Ab 100 nM: n=91; ZEB85 100 nM: n=94; t-test; N=3 independent experiments).
