# Supplementary material for: Actions of the TrkB Agonist Antibody ZEB85 in Regulating the Architecture and Synaptic Plasticity in Hippocampal Neurons
Source: Front Mol Neurosci. 2022 Jun 30;15:945348. doi: 10.3389/fnmol.2022.945348 (PMC9280622; doi:10.3389/fnmol.2022.945348)
Supplement: Supplementary file 2 [file Image_2.pdf]

Tacke et al., Supplemental Figure 2

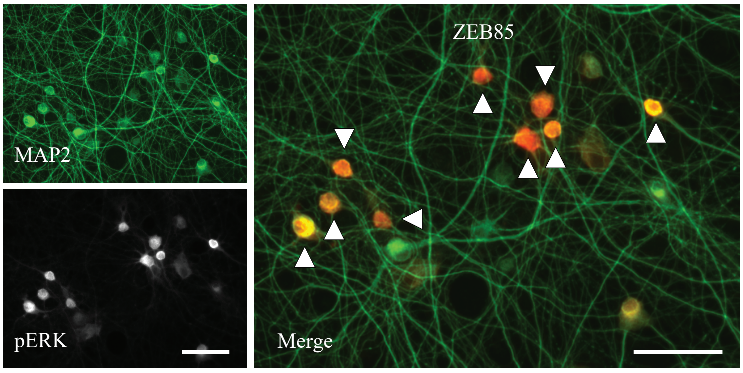

Figure 2 Supplementray: ZEB85 induces cFOS and pERK expression and promotes intracellular calcium dynamics.
Primary hippocampal cultures DIV21 were immunostained for MAP2 (left insert above) and pERK (left insert below). The arrows in the merged image (right) indicate pERK expressing MAP2 positive neurons. Scale bars, 50 µm.
